# Mesenchymal Stem Cell Derived Extracellular Vesicles: A Role in Hematopoietic Transplantation?

**DOI:** 10.3390/ijms18051022

**Published:** 2017-05-09

**Authors:** Luciana De Luca, Stefania Trino, Ilaria Laurenzana, Daniela Lamorte, Antonella Caivano, Luigi Del Vecchio, Pellegrino Musto

**Affiliations:** 1 Laboratory of Preclinical and Translational Research, IRCCS—Referral Cancer Center of Basilicata (CROB), 85028 Rionero in Vulture, Italy; stefania.trino@gmail.com (S.T.); ilaria.laurenzana@crob.it (I.L.); daniela.lamorte@crob.it (D.L.); antonella.caivano@crob.it (A.C.); 2 CEINGE Biotecnologie Avanzate s.c.a r.l., 80147 Naples, Italy; luigi.delvecchio@unina.it; 3 Department of Molecular Medicine and Medical Biotechnologies, University of Naples Federico II, 80138 Napoli, Italy; 4 Scientific Direction, IRCCS—Referral Cancer Center of Basilicata (CROB), 85028 Rionero in Vulture, Italy; pellegrino.musto@crob.it

**Keywords:** mesenchymal stem cells, hematopoietic stem cell transplantation, graft versus host disease, extracellular vesicles

## Abstract

Mesenchymal stem cells (MSCs) are a heterogeneous cellular population containing different progenitors able to repair tissues, support hematopoiesis, and modulate immune and inflammatory responses. Several clinical trials have used MSCs in allogeneic hematopoietic stem cell transplantation (allo-HSCT) to prevent hematopoietic stem cell (HSC) engraftment failure, reduce aplasia post chemotherapy, and to control graft versus host disease (GvHD). The efficacy of MSCs is linked to their immune suppressive and anti-inflammatory properties primarily due to the release of soluble factors. Recent studies indicate that most of these effects are mediated by extracellular vesicles (EVs). MSC-EVs have therefore therapeutic effects in regenerative medicine, tumor inhibition, and immune-regulation. MSC-EVs may offer specific advantages for patient safety, such as lower propensity to trigger innate and adaptive immune responses. It has been also shown that MSC-EVs can prevent or treat acute-GvHD by modulating the immune-response and, combined with HSCs, may contribute to the hematopoietic microenvironment reconstitution. Finally, MSC-EVs may provide a new potential therapeutic option (e.g., transplantation, gene therapy) for different diseases, particularly hematological malignancies. In this review, we will describe MSC and MSC-EVs role in improving allo-HSCT procedures and in treating GvHD.

## 1. Introduction

Allogeneic hematopoietic stem cell transplantation (allo-HSCT) is a choice of treatment for many malignant and non-malignant hematological diseases, but the success of this therapy is limited by several side effects [1,2]. Principal problems are due to the failure in effective eradication of the malignancy, the tolerance of the host, and the onset of infections [1,2]. One of the major complications, with the highest transplant-related mortality rate, is the graft versus host disease (GvHD), an inflammatory immunoreaction against healthy tissues of the patient, induced by donor T-cells and triggered by human leukocyte antigen (HLA) mismatch between the recipient and donor [3].

GvHD may be prevented by several approaches [4]. Steroids are the first-line GvHD treatment and patients who fail the therapy are at high risk to die for GvHD or its related complications. Indeed, there is no common standard treatment strategy for steroid-refractory GvHD patients [5].

Among the most recent therapeutic methods for overcoming all complications related to allo-HSCT and GvHD, mesenchymal stem cells (MSCs) hold a relatively crucial position. MSCs are multipotent progenitor cells with various biological functions including multilineage differentiation, immunosuppression, and tissue-repair promotion [6]. Due to their variegate capacities, MSCs have been widely employed in clinical studies conducted in cardiovascular diseases, type I diabetes mellitus, hepatic cirrhosis, as well as in allo-HSCT and in GvHD (https://clinicaltrials.gov/). Interestingly, recent studies have demonstrated that MSCs release extracellular vesicles (EVs) and have shown that MSC-EVs have similar functions to those of MSCs; as a consequence, MSC-EVs are widely studied as potential therapeutic agents in various diseases [7,8]. In vitro and in vivo studies indicate that MSC-EVs therapy appears to hold substantial promise, particularly in the treatment of immune-based disorders, including complications of allo-HSCT [9,10]. In this article, we will review the characteristics of MSCs and MSC-EVs and their therapeutic role in allo-HSCT and GvHD.

## 2. Definition and Characteristics of MSCs

MSCs are self-renewing and non-hematopoietic cells with multilineage potential to differentiate into cells of mesodermal lineage, like adipocytes, bone, fat, and cartilage cells, as well as into other embryonic lineages [6]. Due to the lack of uniform criteria to define MSCs, in 2006 the International Society of Cellular Therapy established three minimum biological parameters to better identify these cells: (i) plastic adherence in in vitro standard culture conditions; (ii) expression of cluster of differentiation (CD) 105, CD73, and CD90 and no expression of CD45, CD34, CD14 or CD11b, CD79a, or CD19 and HLA-DR (human leukocyte antigen–antigen D related) surface markers; (iii) in vitro differentiation to osteoblasts, adipocytes, and chondrocytes [8,11,12]. MSCs are easily available from different human tissues, such as umbilical cord blood (UCB), bone marrow (BM), adipose tissue, skin, liver, amniotic fluid, and placenta [13]; they can be expanded in vitro by consecutive passaging without significant alteration of their major properties [14]. MSCs release chemokines and cytokines exerting paracrine effects. In BM, MSCs are a relevant component of the hematopoietic stem cell (HSC) niche and support hematopoiesis by their capability to secrete soluble factors, such as stem cell factor (SCF), leukemia inhibitory factor (LIF), and interleukin (IL)-6 [9]. Moreover, they are able to regulate HSC quiescence in the endosteal niche and to control HSC proliferation, differentiation, and recruitment in the vascular niche [6]. Most of the clinical applications involve MSCs from BM that are considered to be safe. In different studies, BM-MSCs are replaced by umbilical cord blood stem cells (UCBsc) that are more primitive and have a higher proliferative capacity than BM-MSCs, thus indicating that they could be a good alternative source in clinical applications [15,16].

## 3. Immune-Regulatory Properties of MSCs 

MSCs are able to exert a wide range of biological functions, prevalently due to their stem/progenitor properties, and immune-regulation and anti-inflammatory abilities. MSCs can influence multiple components of both adaptive and innate immune responses by soluble factors, such as transforming growth factor β (TGF-β), hepatocyte growth factor (HGF), nitric oxide (NO), human leucocyte antigen G (HLA-G), chemokines, and also by cell contact-dependent mechanisms [12]. Numerous studies have shown that MSCs regulate the immune system mainly acting on T- and B-lymphocytes, natural killer (NK) cells, dendritic cells (DCs), monocytes, and macrophages. In particular, MSCs suppress T-cells and favor the maturation of DCs; they reduce B-cell activation and proliferation and inhibit the proliferation and cytotoxicity of NK-cells. Moreover, MSCs promote the generation of regulatory T-cells (Treg), immune system modulators, by the release of soluble factors or by cell–cell contact. Several data report that MSCs suppress T-cell activation and proliferation in response to alloantigens or mitogen stimuli, through the secretion of TGF-β, HGF, and NO [14]. T-lymphocyte suppression, mediated by MSCs, seems to be related to the inhibition of cell cycle division rather than apoptosis induction [17]. MSCs are also involved in the regulation of T-helper1/T-helper2 (Th1/Th2) balance [18]. Recent studies report that MSCs decrease the Th1 response in patients with acute-GvHD (aGvHD) and autoimmune diseases. Other studies report that as a consequence of inflammatory diseases such as allergic rhinitis [19,20] and asthma [21,22], BM-MSCs lead to a shift from Th2 to Th1 response, indicating a variable modulatory effect of MSCs depending on the local microenvironment or disease status [23].

BM-MSCs may suppress immune response by acting on Treg. Aggarwal and Pittenger report that CD4^+^CD25^+^ Treg cells increase after co-culture between peripheral blood (PB) mononuclear cells and MSCs [24,25]. MSCs can also increase the frequency of CD8^+^CD28^−^ T-cells by decreasing apoptosis [26].

MSCs inhibit proliferation and arrest the cell cycle of B-cells [27,28,29]. Conversely, a recent study by Healy et al. reports that human BM-MSCs support the activation, proliferation, and survival of purified CD19+ peripheral B-cells through a cell contact-dependent mechanism [30]. As reported by different studies, MSCs exert an inhibitor effect on NK cells [24,31,32]. Spaggiari et al. show that BM-MSCs are capable of inhibiting the cytokine-induced proliferation of freshly isolated NK cells, but they also prevent NK effector functions, such as cytotoxicity and cytokine production [33]. Moreover, Thomas et al. performed co-culture experiments with BM-derived human MSCs and human NK cells demonstrating that MSCs enhanced the ability of IL-12/IL-18-stimulated NK cells to secrete interferon (IFN)-γ, playing a crucial role in the defense against infections and modulating tissue regeneration [34]. Moreover, MSCs inhibit the differentiation of DCs by inhibiting the expression of major histocompatibility complex (MHC) class II, CD1-α, CD40, CD80, and CD86, and by suppressing proinflammatory cytokine production. This effect prevents the DC mediated activation of T-cells [17,35,36].

Finally, MSCs are able to recruit monocytes and macrophages from across the body and into the inflamed tissue through the release of chemokine (C–C motif) ligands CCL2, CCL3, and CCL12, and promote wound repair [37]. Moreover, the co-culture of human MSCs and monocytes promotes the formation of M2 activated macrophages, which exhibit high levels of IL-10 expression, intense phagocytic activity, low tumor necrosis factor (TNF) and IFN-γ levels, and MHCII expression [38,39]. Monocyte differentiation occurs as a result of cell–cell contact and by several soluble factor-mediated mechanisms, such as indoleamine-pyrrole 2,3-dioxygenase (IDO) and prostaglandin E2 (PGE2) secretion by MSCs [38,39].

## 4. Clinical Applications of MSCs in Allo-HSCT and GvHD

Different studies indicate that MSCs have an important role in modulating the BM microenvironment and supporting hematopoiesis [40,41,42,43]; for these reasons they are widely used in hematological diseases, in particular in allo-HSCT. In clinical practice, MSCs are co-infused with allogeneic HSCs to promote hematological engraftment and prevent engraftment failure and poor graft function [41,44,45]. Published data demonstrate that MSCs may reduce the risk of graft failure by modulating host alloreactivity and/or by promoting a better engraftment of donor hematopoiesis in HLA haploidentical allograft transplantation [41,46,47,48,49]. In UCB transplantation, the co-infusion of MSCs with UCBsc promotes hematopoietic engraftment [50,51] and may have a favorable effect on GvHD prevention [45]. On the contrary, other studies have demonstrated that the co-infusion of MSCs at the time of UCB transplantation has no effect on the kinetics of engraftment and on the prevention of GvHD [52], inhibits thymic reconstitution, and has a negative effect on patient’s survival [53]. MSCs have been also employed to stimulate ex vivo UCB-derived HSC allowed to obtain a great number of total nucleated cells and hematopoietic progenitor cells [54,55]. In a clinical trial conducted on 31 patients receiving two different units of UCBsc, one of which expanded ex vivo with MSCs, a significant improvement of early UCBsc engraftment with respect to the infusion of the un-manipulated UCBsc unit only was shown. However, this study also maintains that the un-manipulated unit provides a long-term engraftment [56]. The clinical trials on the use of MSCs in HSCT are summarized in Table 1, as well as the response rate.

MSCs are also used to treat aGvHD and chronic-GvHD (cGvHD), two major complications that occur after allo-HSCT [57,58,59]. A summary of different clinical trials of MSC therapy for GvHD is provided in Table 2. Overall, these studies have shown that MSC infusion appears to be a safe treatment option for GvHD, not associated with any long-term risk [60,61,62,63,64,65,66,67,68,69,70,71,72,73,74]. Although different studies have confirmed the clinical benefits in patients with steroid resistant grade II–IV aGvHD or cGvHD, other randomized controlled trials are ongoing to assess the definitive efficacy and safety of this treatment modality.

Regarding systemic distribution of infused MSCs, Von Bahr et al. studied different postmortem tissue samples and reported that there are no signs of ectopic tissue formation or MSC-derived malignancies [75]. On the other hand, Forslow et al., in a retrospective study, found that treatment with MSCs may be a risk factor for pneumonia-related mortality after allo-HSCT [76]. Moreover, MSCs co-transplanted with HSCs seem to increase the risk of relapse in patients with hematological malignancies, as reported by Ning et al. [77]. Conversely, another study indicated no significant differences in the rate of leukemia relapse, pulmonary infection, or cytomegalovirus infection within one year after haploidentical HSCT between MSC treated or not treated groups [78]. Finally, studies suggest that the negative response to MSC transfusion in allo-HSCT could be related to the heterogeneity of patient populations treated with different allo-HSCT regimens, different sources of MSCs, severity of aGvHD, and to the use of animal derived products (e.g., fetal bovine serum) in cell culture media [65,66].

## 5. Extracellular Vesicles 

Extracellular vesicles are nanosized, cell-derived particles released by different cell types. Current EVs classification is based on biophysical properties including size, cellular origin, molecular cargo, and biogenesis [79]. The main classes of EVs are exosomes, microvesicles (also referred to as ectosomes or microparticles), apoptotic bodies, and oncosomes [79,80,81,82]. Exosomes are a homogeneous population of vesicles [83] that are released after the fusion of multivesicular bodies with the plasma membrane; their size ranges between 40 and 150 nm [81]. Exosomes are rich in tetraspanins (e.g., CD63, CD81, CD9) [84], gangliosides, phingomyelin, and disaturated lipids that confer a higher rigidity of their lipid bilayer compared with that of cell membranes [79] giving them resistance to degradation and stability as carriers of various biomolecules [85]. Their production is controlled by several regulatory mechanisms, including elements of the endosomal sorting complex required for transport (ESCRT), Rab proteins, tumor protein p53/tumor suppressor activated pathway-6 pathway, ceramide, and neutral sphingomyelinase [84,86,87]. Microvesicles bud directly from the plasma membrane, are generally more heterogeneous in size (50–2000 nm) [81], and contain cytoplasmic cargo [79]. Owing to their origin, microvesicle surface markers are largely dependent on the composition of the membrane from which they originate [9]. Additionally, microvesicles are enriched in a different class of proteins including integrins, glycoprotein Ib (GPIb), and P-selectin [79]. Apoptotic bodies are released upon the fragmentation of cells undergoing apoptosis, their diameters range between 50 and 5000 nm in size, they contain DNA binding histones, and they are depleted in glycoproteins [79,81]. Finally, oncosomes are large EVs (1–10 μm in size) produced by membrane protrusions of malignant cells [79]. Oncosomes carry different bioactive molecules, including signaling factors involved in cell metabolism and metalloproteinases that can digest the extracellular matrix and can contribute to the invasiveness of cancer cells [79]. The number of oncosomes is directly correlated with the aggressiveness of the cancer [88,89]. Oncosomes can alter the homeostasis of the tumor microenvironment by varying the structure and composition of the extracellular matrix or directly by targeting fibroblasts, and endothelial and immune cells [89].

EVs mediate cellular communication by reprogramming target cells [90] and regulating normal physiological processes [91] and pathological conditions [83,92,93,94]. EVs contain genetic and proteomic material from originating cells, thus potentially consisting of a source of potential biomarkers [95,96,97,98]. It has been reported that EVs can be recovered from different biological fluids (e.g., serum, saliva, urine, milk) [93,99,100] suggesting a new perspective for the management of cancer; in fact, they could be used as potential biomarkers by introducing a new concept of “liquid biopsy” [93]. In hematological malignancies, for example, serum EVs are positive for cancer associated surface markers and its number positively correlates with clinical parameters [101,102]. Moreover, EVs are emerging as potent genetic information agents supporting a range of biological processes and with therapeutic potential [93].

## 6. Characteristics and Clinical Applications of MSC-Derived EVs

It is well known that MSCs release EVs [103]. MSC-derived EVs conserve the common specific exosomal surface markers, such as CD107, CD63, CD9, and CD81 [104]. They also express surface markers which are characteristic of their cells of origin, such as CD29, CD73, CD44, and CD105 [9]. Several studies have analyzed their content of nucleic acid and proteins which is transferred to the target cells. Tomasoni et al. demonstrated that MSC-derived EVs contain several classes of RNAs, in particular, transcripts involved in the control of transcription (transcription factor CP2, clock homolog), cell proliferation (retinoblastoma-like 1, small ubiquitin-related modifier 1), and immune regulation (interleukin 1 receptor antagonist) [105]. Additionally, MSC-derived EVs contain specific microRNAs, such as miR-223, miR-564, and miR-451, involved in multi-organ development, cell survival, differentiation, and immune-regulation [104,106]. Characterization of MSC-derived EVs content reveals the abundance of several proteins. Interestingly, proteome analysis shows the presence of cytoplasmatic proteins such as surface receptors (PDGFRB, β-type platelet-derived growth factor receptor; EGFR, epidermal growth factor receptor; PLAUR, plasminogen activator urokinase receptor), signaling molecules (RRAS/NRAS, RAS-related protein/neuroblastoma RAS; MAPK1, mitogen-activated protein kinase 1; GNA13/GNG12, guanine nucleotide-binding protein subunit α-13/G protein subunit γ 12; Cdc42, cell division control protein 42 homolog; VAV2, Vav guanine nucleotide exchange factor 2), and cell adhesion molecules (FN1, fibronectin 1; EZR, ezrin; IQGAP1, IQ motif containing GTPase activating protein 1; CD47; integrins; LGALS1/LGALS3, lectin galactose binding soluble 1/lectin galactose binding soluble 3). Functional enrichment analysis shows that cellular processes, represented by the MSC-EV proteins, include cell proliferation, adhesion, migration, and morphogenesis, but also self-renewal and differentiation (TGF-β, transforming growth factor beta; MAPK, mitogen-activated protein kinase; PPAR, peroxisome proliferator-activated receptor) [107]. EVs, by using their content, mediate intercellular communication and interact with target cells influencing fundamental biological functions.

Recent studies have shown that MSC-EVs may represent a novel “acellular” therapeutic approach in regenerative medicine [108]. In fact, MSC-EVs may play a role in local tissue repair influencing progenitor cell proliferation, recruitment, and differentiation; promoting extracellular matrix remodeling and angiogenesis; and overcoming apoptosis and immunological responses. Moreover, they have an important function in stem cell plasticity and tissue regeneration, possibly contributing to the paracrine action observed upon MSCs cell transplantation [108]. MSC-exosomes could be used as a novel therapeutic modality, i.e., for cardiac diseases because they protect against acute tubular injury and reduce myocardial ischemia/reperfusion damage [109]. In addition to cardioprotective effects, MSC-EVs may represent a potential therapeutic approach to kidney diseases due to their capability of reducing fibrosis, tubular atrophy/apoptosis, and regenerating tubuloepithelial tissue [7]. Moreover, several in vitro and in vivo studies demonstrate that MSC-EVs therapy potentially promotes liver regeneration following acute injury by directly enhancing hepatocyte survival and proliferation [110].

Instead, in the tumor development context MSC EVs have a controversial role, as widely discussed by Lopatina et al. [111]. Interestingly, they report that MSC-EVs can promote or inhibit tumor growth, indicating that these different effects are probably due not only on the type and stage of the tumor, but also on the MSC culture conditions that may modify the cell secretome [111]. EVs from MSCs could be also used for drug delivery. For the first time, Pascucci et al. demonstrated that following priming with paclitaxel, MSCs are able to strongly inhibit pancreatic tumor thanks to their ability to package and deliver active drugs through microvesicles which are taken up by the cancer cells [112].

## 7. Therapeutic Power of MSC-EVs in Allo-HSCT and GvHD

**Figure 1:**
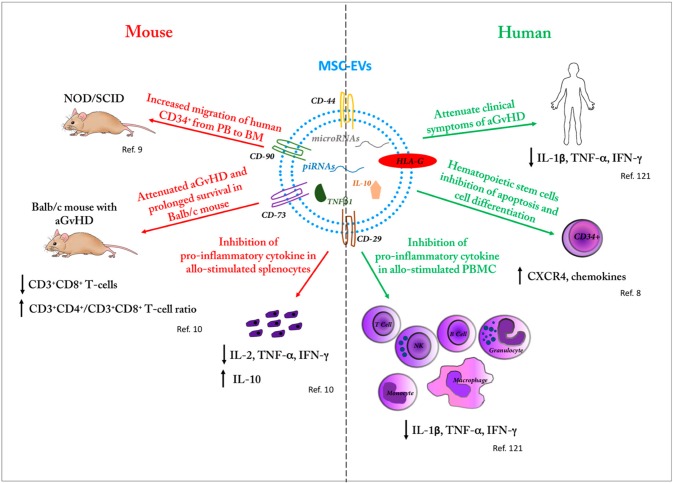
Scheme illustrating the therapeutic power of mesenchymal stem cells extracellular vesicles (MSC-EVs) in HSCT and GvHD. The reported MSC-EVs effects in mice (in vivo and in vitro) are in red. The reported MSC-EVs effects in human (in vivo and in vitro) are in green. PB: Pheripheral blood; PBMC: Pheripheral blood mononuclear cells.

Recent in vivo experiments suggest that the use of MSC-EVs may contribute to improving allo-HSCT. We studied the interaction between UCBsc and BM-MSC-EVs, demonstrating their cross talk and providing a new insight into the biology of cord blood transplantation [9]. In particular, by sequencing MSC-EVs small RNAs, we identified 87 miRNAs and 5 Piwi-interacting RNAs (piRNAs) able to modify the UCBsc fate. In fact, we demonstrated that CD34^+^ cells from UCBsc, exposed to EVs, significantly change different biological functions, becoming more viable and less differentiated. Moreover, EVs treatment of UCBsc induced an increase of C–X–C chemokine receptor type 4 (CXCR4) expression, a key component of the HSC niche, as well as an in vivo augmented migration of CD34^+^ cells from the PB to BM niche (Figure 1) [9]. In another study, murine BM-MSC-EVs treatment induced the loss of quiescence and expansion of murine hematopoietic progenitor cells [113]. The proliferation was mediated via the myeloid differentiation primary response 88 (Myd88) adapter protein and by toll-like receptor 4 [113].

Due to their capacity to modulate immune response [114,115,116,117,118,119,120], MSC–EVs could be used to attenuate an activated immune system or to prevent immunoreactions such as GVHD. In this setting, Kordelas et al. demonstrated that BM-MSC-EVs were able to alleviate symptoms in a treatment-resistant, grade IV aGvHD patient, who remained stable for five months after MSC-EVs therapy. They also tested in vitro MSC-EVs containing anti-inflammatory cytokines (IL-10, TGF β, and HLA-G) on PB mononuclear cells and NK cells isolated from one patient that were stimulated with allogeneic target cells; such a treatment resulted in a decreased release of pro-inflammatory cytokines (IL-1β, TNF-α, and IFN-γ) [121]. Moreover, in vivo MSC-EVs therapy reduced the pro-inflammatory cytokine response and the clinical symptoms of GvHD. Additionally, MSC-EVs infusion was well tolerated and no side effects were reported (Figure 1) [121].

Wang et al. instead showed that UCB-MSC-EVs could prevent aGvHD in a mouse model of allo-HSCT by modulating immune responses [10]. The study demonstrated that UCB-MSC-EVs reduce in vivo manifestations of aGvHD, attenuate the associated histological changes, and prolong mice survival. In fact, in recipient mice a significant decrease of frequency and the absolute number of CD3^+^CD8^+^ T-cells and an increased ratio of CD3^+^CD4^+^ to CD3^+^CD8^+^ T-cells were found [10]. Finally, as reported by Kordelas et al. [121], in vitro and in vivo experiments confirmed the decreased levels of different inflammatory cytokines, including IL-2, TNF-α, and IFN-γ, and an increase of anti-inflammatory cytokines, such as IL-10 (Figure 1) [10].

## 8. Conclusions

Nowadays, MSCs are excellent candidates for improving the clinical therapeutic potential of HSCT and controlling GvHD. Preclinical and clinical results clearly show the efficacy of MSC treatment in the majority of studies. However, there are still many inconveniences, such as the increased risk of pneumonia-related death after allo-HSCT [76], the uncontrolled differentiation, and the unwanted long-term side effects [122]. Moreover, standardized techniques of MSCs production are still missing.

In this context, MSC-EVs, when compared with MSCs, seems to have several advantages. In particular, EVs appear to be safer than MSCs [121,123], allowing them to overcome at least some of the aforementioned problems concerning MSC clinical applications. Moreover, compared to cells EVs are more stable and reversible, have no risk of aneuploidy and a lower possibility of immune rejection due to their small size, and lower expression of membrane-bound molecules, including histocompatibility molecules [124,125]. MSC-EVs protect their contents from in vivo degradation, thus preventing problems associated with the rapid break down of soluble molecules. Overall, they provide a very promising alternative therapy in the context of allo-HSCT [124]. However, the development of standardized procedures for the isolation and storage of EVs is still needed, as well as the improvement of methods and criteria for the quality analysis of EV-based therapies.

## Figures and Tables

**Table 1:** Clinical trials of mesenchymal stem cells (MSC) application to promote hematopoietic stem cell (HSC) engraftment in phase I/II.

Clinical Studies	MSC Source	No. of pts	Outcome	References
Breast cancer; autologous HSCT	BM	28	Rapid hematopoietic recovery	Koc et al.
		adults		[46]
Hematological malignancy; autologous HSCT	BM	162	Improvement of early lymphocyte recovery	Batorov et al.
		adults		[48]
Hematological malignancy; allogeneic HSCT	BM	46	Prompt hematopoietic recovery	Lazarus et al.
		adults		[43]
Hematological disorders; haplo-T-cell-depleted HSCT	BM	14	Accelerate leukocyte recovery. Prevention of graft rejection	Ball et al.
		children		[40]
Hematological disorders; UCBT	BM	8 children	Prompt hematopoietic recovery	Macmillan et al. [50]
Hematological disorders; UCBT	BM	13 children	No effect on engraftment and hematopoietic recovery. GvHD prevention	Gonzalo-Daganzo et al. [51]
Hematological disorders; UCBT+ 3rd party HSCs	BM	9	No effect on engraftment and GvHD	Bernardo et al. [44]
		adults		
Severe aplastic anemia	UCB	21	Sustained donor engraftment	Wu et al.
		adults		[47]
Hematological malignancy; allogeneic HSCT	UCB	50	Sustained donor engraftment	Wu et al.
		adults and children		[45]

pts: Patients; HSCT: Hematopoietic stem cell transplantation; BM: Bone marrow; UCBT: Umbilical cord blood transplantation; GvHD: Graft versus host disease; UCB: Umbilical cord blood.

**Table 2:** Clinical trials of MSC for the treatment of GvHD.

Clinical Studies	MSC Source	No. of pts	Outcome	References
Grade II–IV aGvHD after allogeneic HSCT/DLI	BM	55	OR: 70%; CR: 54%; improved OS in responders	Le Blanc et al.
		adults and children		[59]
Grade IV aGvHD after allogeneic HSCT	BM	1	Complete resolution of grade IV aGvHD	Le Blanc et al.
		children		[60]
Grade III–IV aGVHD and extensive cGVHD after allogeneic HSCT	BM	9	5 pts with aGvHD survived 2 months to 3 years after HSCT	Ringden et al.
		adults	Transient response in the liver, but not in the skin in cGvHD	[61]
Grade II–IV aGvHD after allogeneic HSCT/DLI	BM	31	CR: 77%; PR: 16%	Kebriaei et al.
		adults		[62]
Grade III–IV aGvHD after allogeneic HSCT/DLI	BM	13	2 pts (15%) responded and required no further immunosuppressant therapy.	Von Bonin et al.
		adults	OR, 28 days after first MSC infusion, was 54%	[64]
Grade III aGvHD after allogeneic HSCT	BM	3	CR: 33%; PR: 67%	Arima et al.
		adults		[65]
Grade II–IV aGvHD after allogeneic HSCT	Adipose tissue	6	CR: 83%	Fang et al.
		adults		[66]
GVHD after allogeneic HSCT	BM	7	2 pts with severe aGvHD did not progress to cGvHD. 1 out of 3 pts showed slight improvement of cGVHD	Muller et al.
		children		[67]
Grade II–IV aGvHD after allogeneic HSCT/DLI	BM	11 children	OR: 71%; CR: 24%	Lucchini et al.
				[63]
Grade II–IV aGvHD after allogeneic HSCT/DLI	BM	37 children	CR: 59%; improved OS especially in early MSC treatment	Ball et al.
				[68]
Grade II–IV aGvHD after allogeneic HSCT/DLI	BM	40	OR: 67%; CR: 27%. Better in children and grade II	Introna et al.
		adults and children		[69]
Extensive sclerodermatous cGVHD after allogeneic HSCT	BM	4	Improvement in signs of cGVHD	Zhou et al.
		adults		[70]
Refractory cGVHD after allogeneic HSCT	BM	23	OR: 87%. Increase in Bregs	Peng et al.
		adults		[71]
Grade III–IV aGvHD after allogeneic HSCT	BM	25	CR: 24%; OS: 60%	Muroi et al.
		adults		[72]
Grade I–IV aGvHD after allogeneic HSCT/DLI	BM	58	OR: 47%, CR: 9%	Von Dalowski et al.
		adults		[73]

MSC: Mesenchymal stem cell; pts: Patients; aGVHD: Acute-graft versus host disease; HSCT: Hematopoietic stem cell transplantation; DLI: Donor lymphocyte infusion; BM: Bone marrow; OR: Overall response; CR: Complete response; OS: Overall survival; PR: Partial response; cGVHD: Chronic-graft versus host disease; Bregs: Regulatory B-cells.
